# Supplementary material for: Motor cortical output for skilled forelimb movement is selectively distributed across projection neuron classes
Source: Sci Adv. 2022 Mar 9;8(10):eabj5167. doi: 10.1126/sciadv.abj5167 (PMC8906739; doi:10.1126/sciadv.abj5167)
Supplement: Supplementary file 1 — Figs. S1 to S12 [file sciadv.abj5167_sm.pdf]

Science Advances
AAAS

Supplementary Materials for

Motor cortical output for skilled forelimb movement is selectively distributed across projection neuron classes

Junchol Park*, James W. Phillips, Jian-Zhong Guo, Kathleen A. Martin, Adam W. Hantman, Joshua T. Dudman*

*Corresponding author. Email: yojcpark@gmail.com (J.P); dudmanj@janelia.hhmi.org (J.T.D.)

Published 9 March 2022, *Sci. Adv.* **8**, eabj5167 (2022)
DOI: 10.1126/sciadv.abj5167

The PDF file includes:

Legends for movies S1 to S3
Figs. S1 to S12

Other Supplementary Material for this manuscript includes the following:

Movies S1 to S3

Supplemental video:

Supplementary video 1. Example trials with/without closed-loop inactivation of $MCtx^{FL}$ neural activity by activation of inhibitory neurons in VGAT-ChR2 mice.
(Left) A representative trial with closed-loop inactivation of $MCtx^{FL}$. A filled circle in the upper left corner indicates frames with laser on. Numbers in the bottom left corner indicate time relative to the laser onset triggered by slight movement of the animal. Video replay is at x0.2 speed.
(Right) A representative trial without closed-loop inactivation. An empty circle in the upper left corner indicates frames for which the laser would have been delivered. Numbers in the bottom left corner indicate time relative to the pseudo laser onset.
Video 1 can be found online at:
https://www.dropbox.com/s/vy6dzrqwm4w1eix/Trial%2354_139_stim-Pstim%20%28Converted%29.mov?dl=0

Supplementary video 2. Inactivation of IT neurons triggered by the hand lift discontinues an ongoing reaching.
The video provides dual views on a mouse performing a reach-to-grasp task. To highlight the necessity of continuous IT neuronal dynamics for a successful forelimb reach, we automatically triggered a laser at the detection of a voluntary hand lift, which inactivated IT neurons using the inhibitory opsin GtACR2. The laser onset is signaled by a LED light (invisible to the mouse) in the video. IT inactivation discontinued the ongoing reaching and blocked subsequent reaching during inactivation which lasted for 2 s.
Video 2 can be found online at:
https://www.dropbox.com/s/1sayuypoujj6ir1/IT_liftTriggeredSilencing_20200125_v007.mp4?dl=0

Supplementary video 3. Inactivation of PT neurons of $MCtx^{FL}$ has little impact on forelimb movement in a reach-to-grasp task.
The current trial begins with a cue signaling placement of the pellet in the target position which coincided with the onset of a laser that inactivated pons-projecting PT neurons using the inhibitory opsin GtACR2. The cue/laser onset is signaled by a LED light. PT inactivation had little impact on reaching.
Video 3 can be found online at:
https://www.dropbox.com/s/rgpppdmlid12qea/PT_silencing_20200426_v120.mp4?dl=0

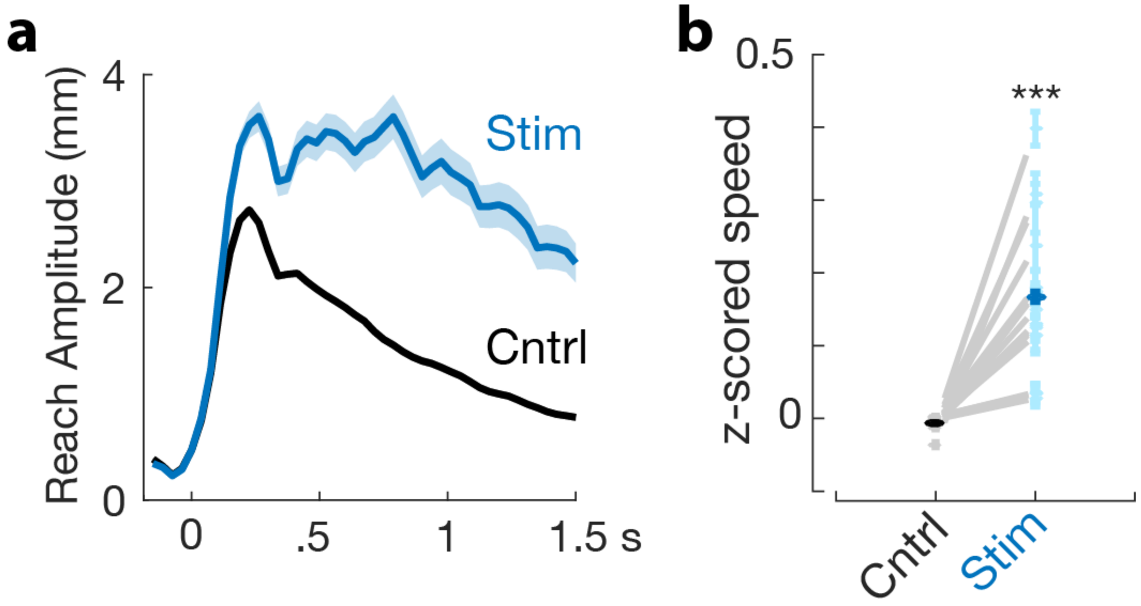

Fig. S1. Stimulation of layer 5 output neurons in MCtx^{FL} invigorates forelimb movement
Closed loop stimulation of the majority of descending layer 5 output neurons in MCtx^{FL} labelled using the Rbp4-cre line (47) crossed to Ai32 (74) produced increases in the amplitude (*top*) and speed (*bottom*) of forelimb movement.

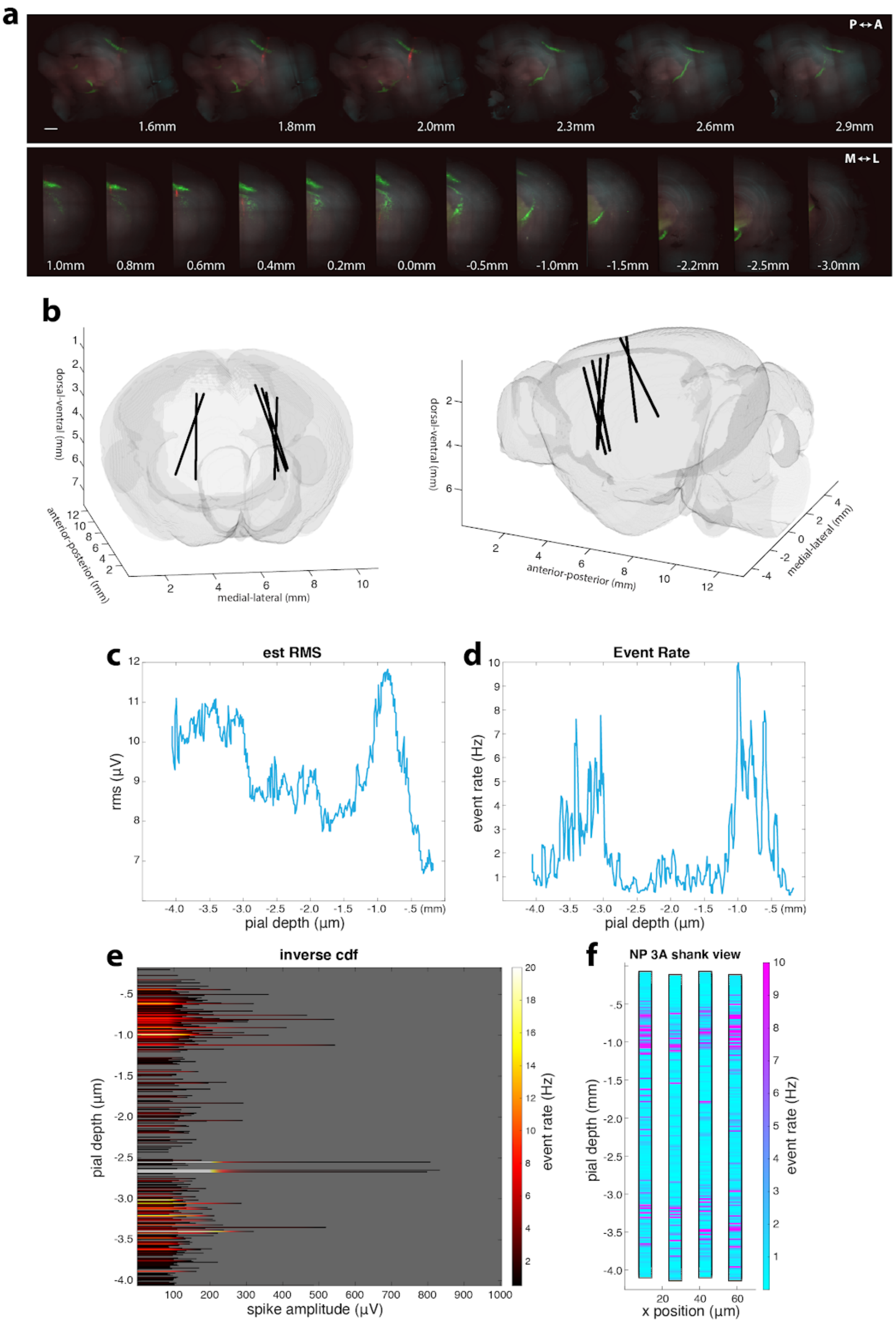

a
P↔A
1.6mm
1.8mm
2.0mm
2.3mm
2.6mm
2.9mm
M↔L
1.0mm
0.8mm
0.6mm
0.4mm
0.2mm
0.0mm
-0.5mm
-1.0mm
-1.5mm
-2.2mm
-2.5mm
-3.0mm
b
dorsal-ventral (mm)
anterior-posterior (mm)
medial-lateral (mm)
c
est RMS
rms (µV)
pial depth (µm)
d
Event Rate
event rate (Hz)
pial depth (µm)
e
inverse cdf
pial depth (µm)
spike amplitude (µV)
event rate (Hz)
f
NP 3A shank view
pial depth (mm)
x position (µm)
event rate (Hz)

Fig. S2. Histological verification of Neuropixels recordings

a, A sagittal (top) and coronal (bottom) view of a cleared mouse hemi brain imaged with light sheet microscopy. Green fluorescence indicates labeling of the deep layer 5 PT neurons and their projections to downstream areas such as striatum, superior colliculus and pons. Red fluorescence indicates probe tracks. Numbers in the top and bottom rows indicate medial-lateral and anterior-posterior coordinates relative to bregma, respectively. The length of the white scale bar = 1mm. **b**, Three dimensional rendering of probe tracks with the Allen Anatomical Template (AAT). Imaged 3D brain volumes were aligned to AAT, and each electrode on Neuropixels probe was assigned to a brain region using the probe geometry and Allen Reference Atlas (Methods). All cells recorded from electrodes located at the pial depth of 1.75 mm or upper (estimated by the manipulator) were assigned a motor cortical region. **c**, Estimated root mean square voltage (RMS) is plotted versus the pial depth. Note the trough indicating dearth of neural activity around the depth of 1.75 mm, which is consistent with the histologically estimated cortical border. **d**, Mean event rate is plotted versus the pial depth. An event is defined as voltage crossing (e.g. spikes) a threshold (80 μV). Note the elevated event rates of the cortical depths. **e**, Threshold-crossing events are binned and counted based on the absolute raw amplitude for each pial depth. **f**, Mean event rates are plotted for each electrode site tiling the Neuropixel probe. Note the higher event rates within the cortical range as well as the dearth of events around the histologically estimated cortical border (1.75 mm). RMS and event rates were measured using codes written by Jennifer Colonell (https://github.com/jenniferColonell/Neuropixels_evaluation_tools).

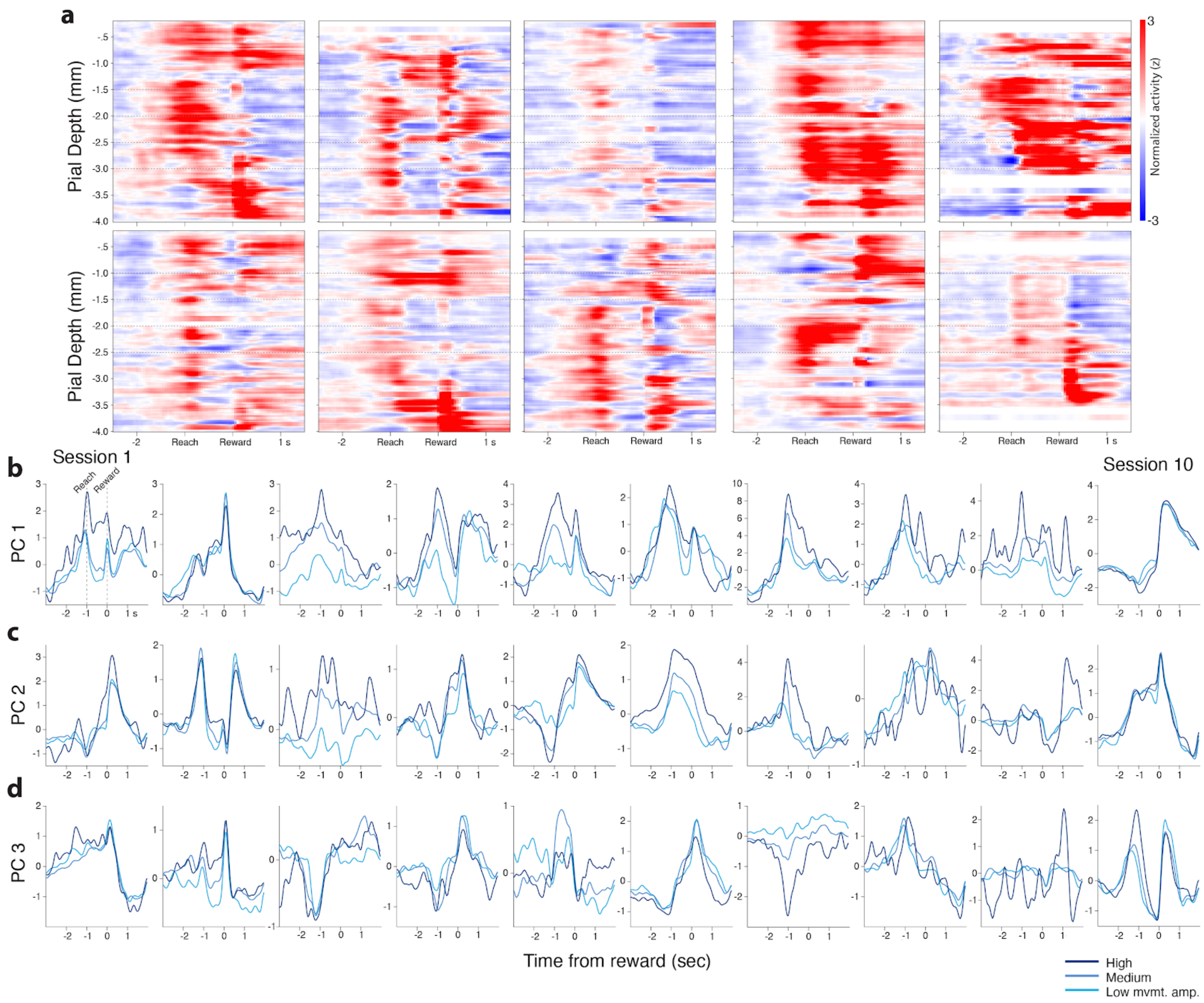

Fig. S3. Summary of cortical and striatal neural population activity during task performance
a, Mean z-score normalized activity aligned to reach threshold crossing and reward delivery (x axis) is plotted per inferred depth (y axis) relative to the pial surface for all individual recording sessions (N=10). **b-d**, MCtx^{FL} neural population activity of movement amplitude tertiles are projected onto the top three principal components. Neural trajectories indicate that at least partially separable populations of neurons are active during forelimb movements scaling with reach amplitude. Other populations appear to be active during reward collection.

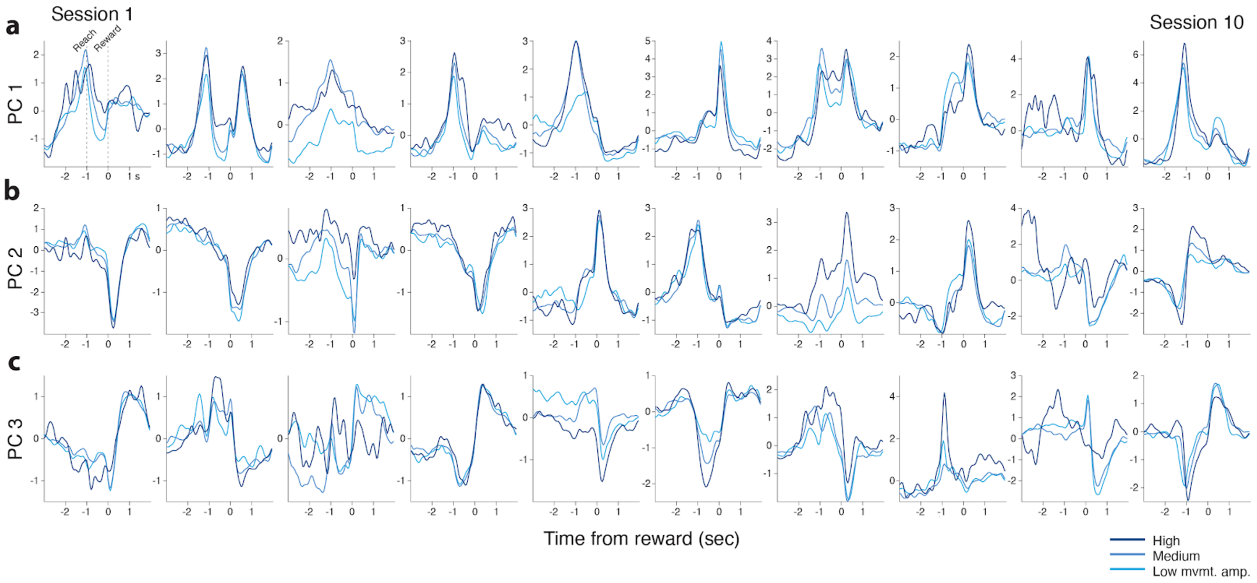

Fig. S4. Projection of striatal neural population activity onto top three PCs
a-c, Striatal neural population activity of movement amplitude tertiles are projected onto the top three principal components. Similar to the cortical data in Fig. S4, neural trajectories indicate that at least partially separable populations of neurons are active during forelimb movements or reward collection.

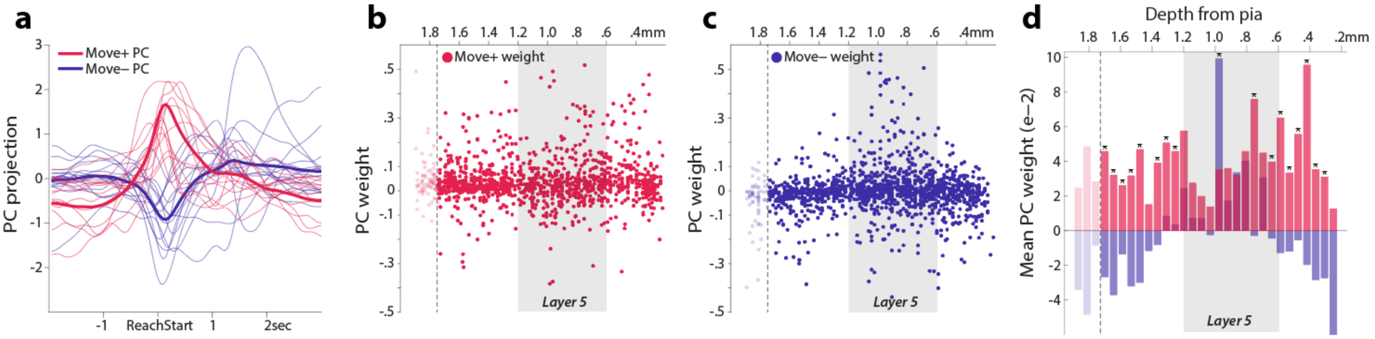

Fig. S5. Laminar inhomogeneity of movement-related activity
a, MCtx^{FL} neural activity projected onto the top three PCs (Methods). PCs were classified as 'Move+', if the neural trajectory on each PC dimension (PC score) peaked before +500 ms relative to the reach start. Other PCs that peaked afterwards with negative modulation during reach were classified as 'Move-'. **b**, Weights of move+ PCs are plotted as a function of recording depth across all cortical neurons (solid circles). A dotted line indicates the histologically-verified cortical border (1.75 mm, see Methods and fig. S3). **c**, Weights of move- PCs are plotted as a function of recording depth. **d**, Weights of move+ and move- PCs significantly differ across cortical depths (Two-way ANOVA, $F_{1,29}$ = 2.15, p = 4.0x10^{-4}, bin-by-bin test, p < 0.05).

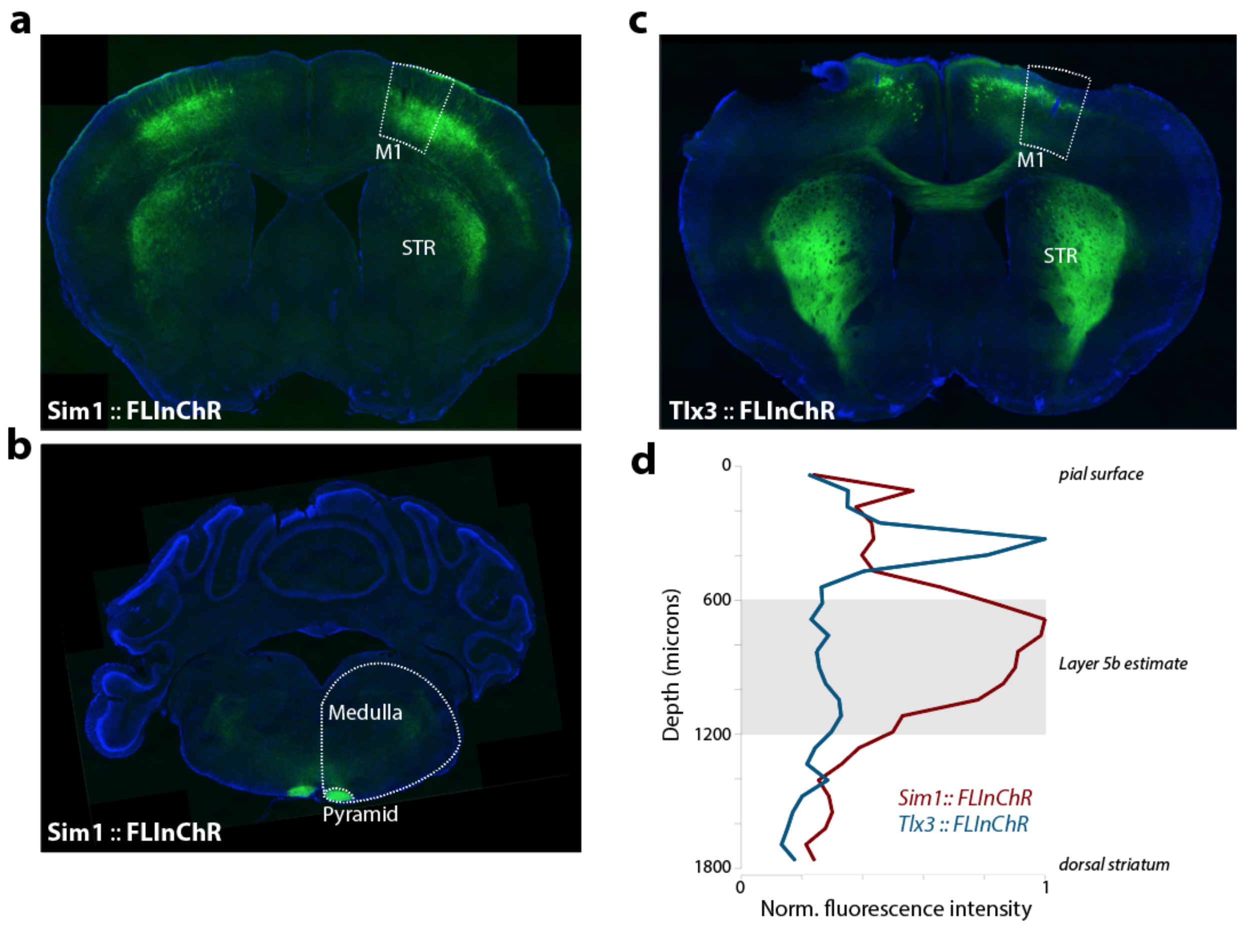

Fig. S6. Histological verification of PT and IT neuronal labelling
a, Expression of fluorescently-labelled FLInChR in the deep layer 5 PT neurons of Sim1-cre mice. **b**, Fluorescent labelling of the medullary pyramid in PT mice. **c**, Expression of FLInChR in the layer 5 IT neurons of Tlx3-cre mice. **d**, To compare the laminar distribution of PT and IT neurons in the motor cortex, the intensity of fluorescence was measured along a line oriented from the pial surface through M1 to the dorsal surface of STR. This distance was normalized to 1800 microns which is our estimate from the allen mouse brain atlas. The fluorescence intensity data were binned into 250 evenly spaced bins and averaged within each bin. The binned data were normalized to max intensity for each cell type.

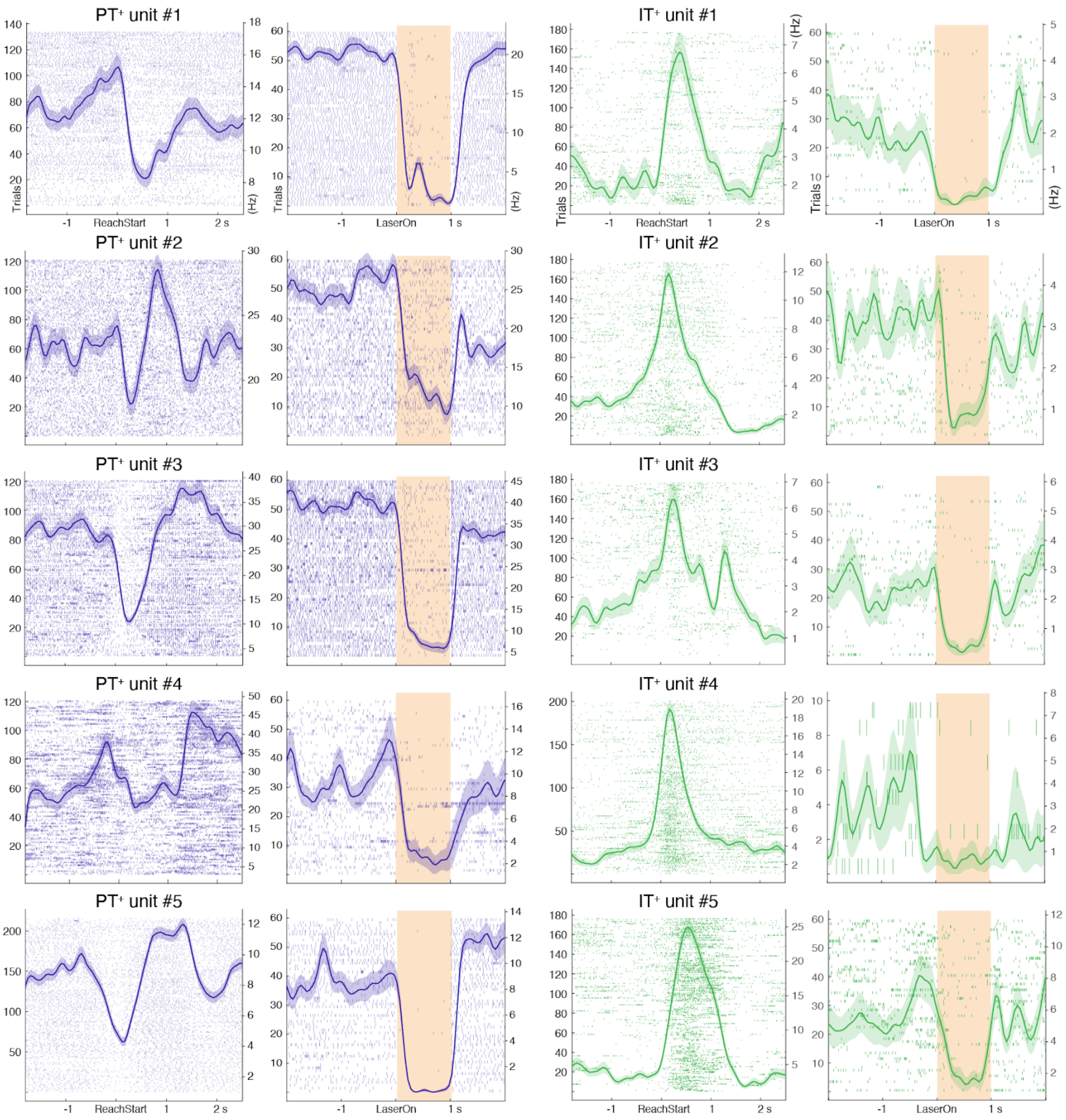

Fig. S7. PT⁺ and IT⁺ neuronal activity during task performance and optotagging
Blue-colored rasters in the left column illustrate trial by trial individual neuronal responses during task performance (left panel of each pair, aligned to reach start) with significant inhibitory responses during optotagging (right panel of each pair, aligned to laser onset) in Sim1-Cre (KJ18Gsat) mice injected with rAAV2-retro-CAG-Flex-FLInChR-mVenus to the pons. Each row represents each trial. The mean±SEM spike rate (Hz) is superimposed. Numbers on the left and right ordinates of each plot indicate the number of trials and firing rate in Hz, respectively. Green-colored rasters in the right column illustrate trial by trial IT⁺ individual neuronal responses during task performance (left panels) and optotagging (right panels) in Tlx3-Cre (PL56Gsat) mice.

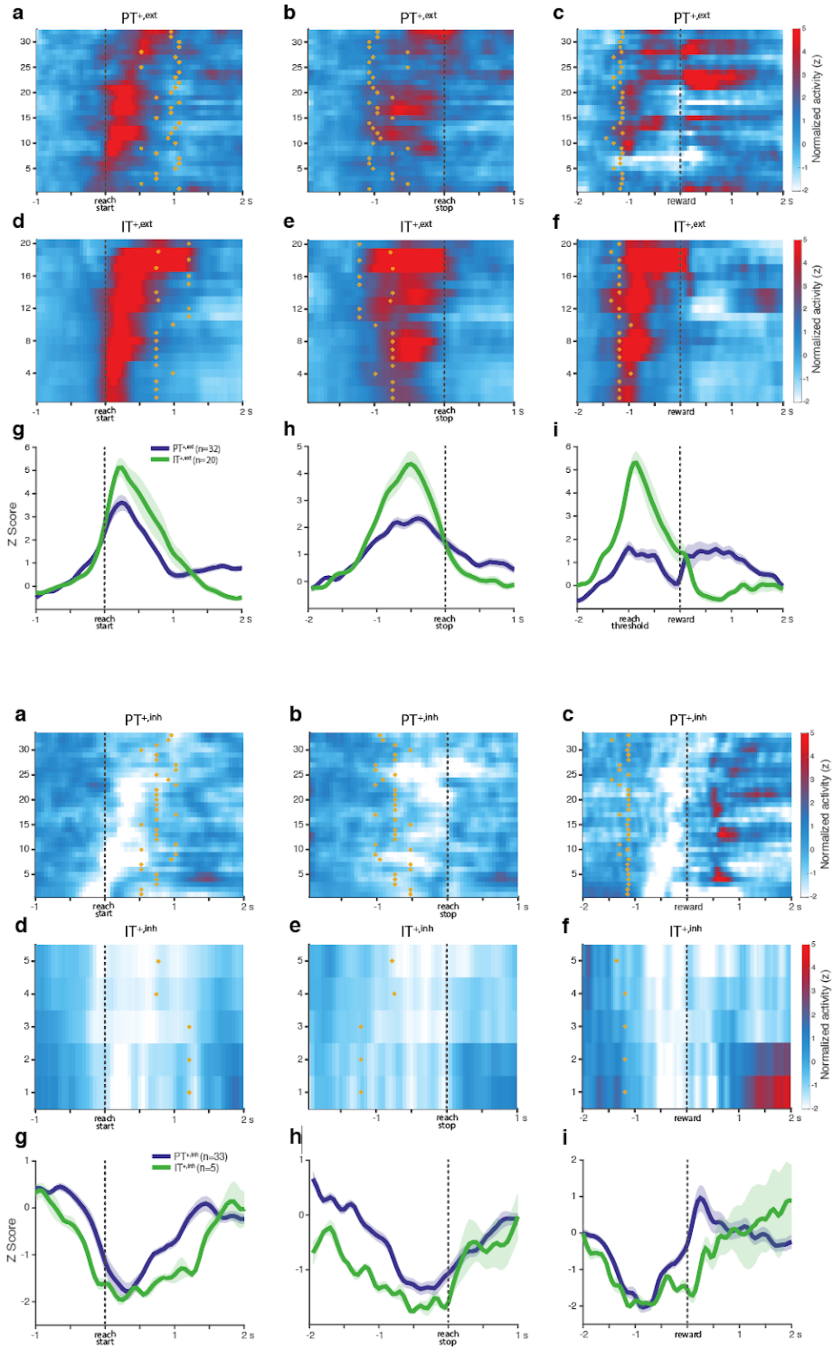

Fig. S8. Activity of PT^{+} and IT^{+} neurons with a significant positive modulation of activity during movement aligned to different movement phases

Upper: **a**, The mean z-score normalized activity of $PT^{+,ext}$ neurons (n=32 of 111 PT^{+}) aligned to reach start. To indicate the mean duration of movement an asterisk was superimposed on each row at the time bin where reach stop occurred. **b**, Activity of the same $PT^{+,ext}$ neurons but aligned to reach stop. Asterisks indicate the mean time of reach start. Neurons were identically sorted along the y-axis across panels (a-c). **c**, Activity of the same $PT^{+,ext}$ neurons but aligned to reach threshold crossing and reward delivery. Note that there was a constant 1-sec delay between the reach threshold crossing and the reward delivery. Asterisks indicate the time of reach start. **d**, Activity of $IT^{+,ext}$ neurons (n=20 of 30 IT^{+}) aligned to reach start. **e**, Activity of $IT^{+,ext}$ neurons alignmed to reach stop. **f**, Activity of $IT^{+,ext}$ neurons alignmed to reach threshold crossing and reward delivery. **g-i**, For comparison, the mean±SEM normalized activities of $PT^{+,ext}$ and $IT^{+,ext}$ neurons were plotted with alignment to different movement phases. *Lower:* same as Upper but for negative modulation around movement.

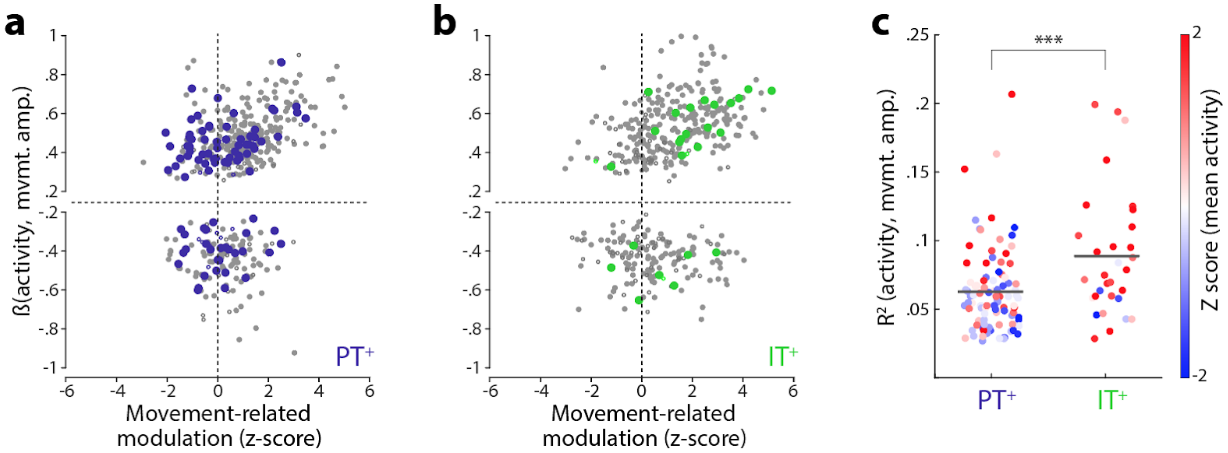

Fig. S9. Cell-type specific individual neuronal correlation with movement amplitude

a, Individual PT⁺ units are plotted based on their normalized movement-timed activity along X axis and their activity modulation as a function of movement amplitude along Y axis (regression coefficients, β). For instance, a unit in the first quadrant is the one that increased its firing rate during movement with a positive correlation with the movement amplitude. Blue-filled circles represent PT⁺ units with a significant regression coefficient (*t* test, α=0.05). Gray circles represent the rest of $MCtx^{FL}$ units that were not tagged. Fisher's exact test against a uniform distribution of PT⁺ units across quadrants, p=0.84). **b**, The vast majority of the individual IT⁺ units located in the first quadrant (Fisher's exact test, p=0.0056). Green-filled circles represent IT⁺ units with a significant regression coefficient. Gray circles represent the rest of $MCtx^{FL}$ units that were not tagged. **c**, Squared Pearson correlation between normalized firing rate and reach amplitude are plotted for individual PT⁺ and IT⁺ units color-coded by their mean movement-timed activity.

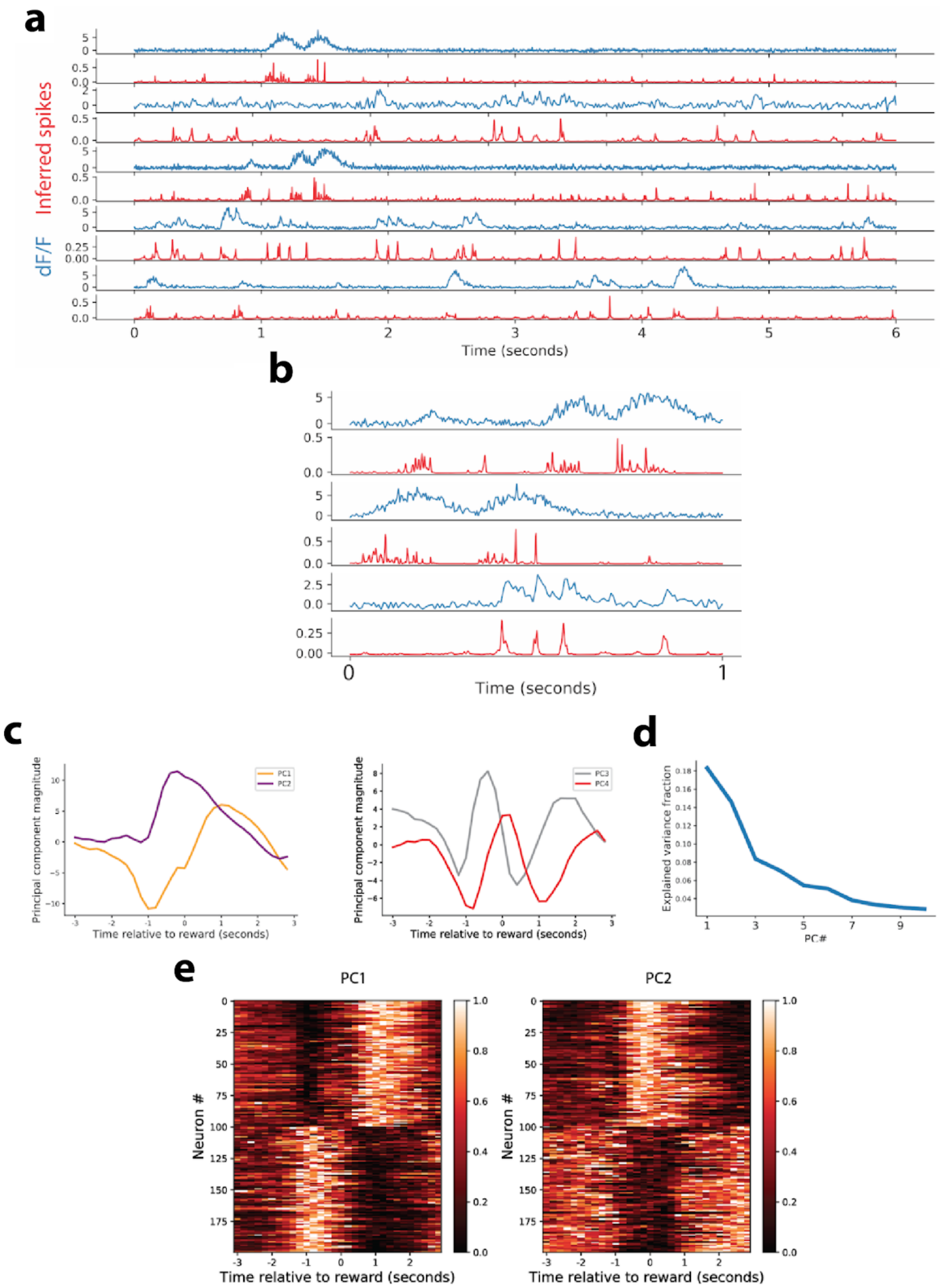

Fig. S10. Spike deconvolution and PCA for calcium imaging data.
a, Illustration of spike deconvolution. Panel shows 5 example regions of interest, each of two rows. Top row for each unit (blue) shows the dF/F trace, with the row beneath (red) showing the inferred spike activity metric. **b**, a zoomed-in portion for three units from **a**. **c**, Structure of first four principal components of neural activity across all units in the dataset, aligned to reward. **d**, The fraction of explained variance for the top 10 principal components in the dataset. **e**, *left*, The normalized inferred spike rates of representative units with most positive (top 100 rows) and negative (bottom 100 rows) weights for PC1 are plotted. Format same as Fig. 3e. *right*, The normalized inferred spike rates of representative units with positive (top 100 rows) and negative (bottom 100 rows) weights for

PC2 are plotted.

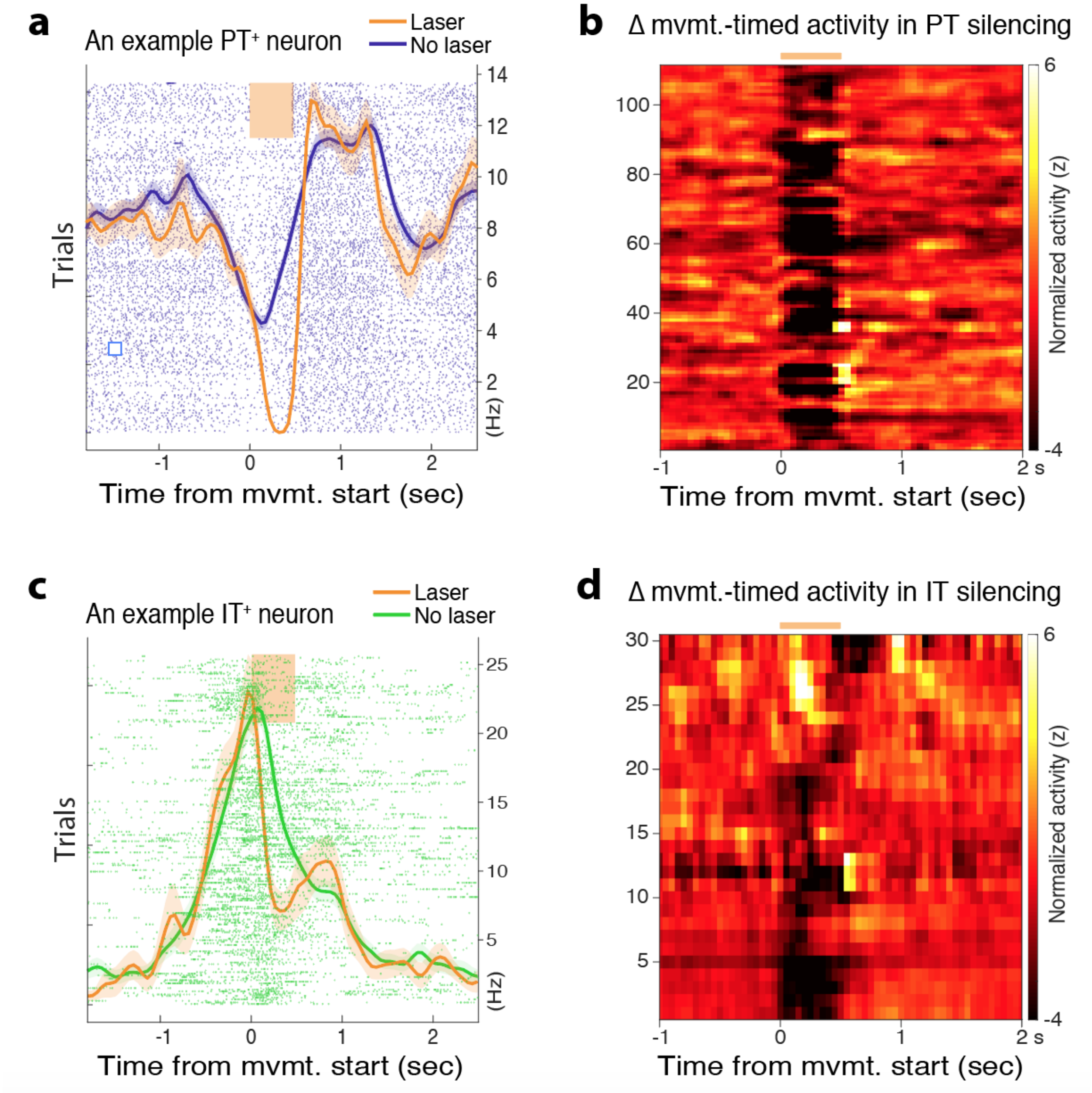

Fig. S11. Robust PT⁺ and IT⁺ neuron inactivation during task performance
a, An example PT⁺ neuron displays a robust inactivation by the laser triggered at the earliest detection of reach in randomly selected trials (top raster rows: laser trials; bottom raster rows: control trials). The mean±SEM spike density (Hz) functions are superimposed for laser and control trials. **b**, Change of all individual PT⁺ neuronal activity by opto-silencing aligned to the movement onset. **c**, An example IT⁺ neuron displays a robust inactivation by the laser. **d**, Change of all individual IT⁺ neuronal activity by opto-silencing.

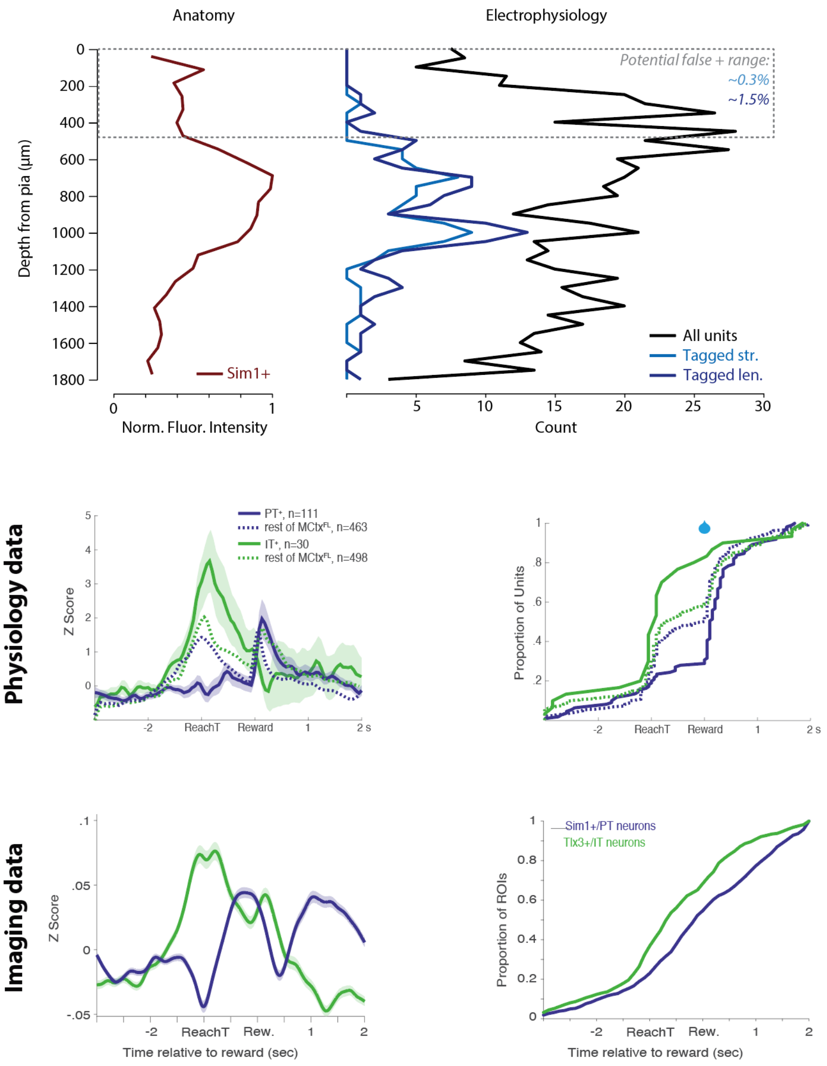

Fig. S12. Comparing anatomical distribution of cells with putative identified cell types from optotagging

These datasets are almost certainly still dominated by false negatives - individual units that are representative of a given cell type, but fail to meet inclusion criteria from optotagging. However, there is also the possibility that a given unit shows strong modulation during the tagging procedure (inhibition to illumination consistent with expression of FLInChR). Our most stringent ('str.') inclusion criteria include mean suppression of firing below -0.3 z score units (z score defined over entire recording duration) sustained for the entire duration of illumination (based upon evidence that FLInChR can produce non-desensitizing inhibition for >500 ms (*70*)). Our more lenient criterion did not require sustained inhibition for the entire illumination duration ('len.'). All results based upon tagged units in the paper are confirmed to be consistent over a range of stringencies. We consider the false positive rate at this stringency which is used for results from key behavior-physiology sessions used for comparison of cell type differences in encoding in Figure 4 in particular. Here we align estimates of PT (Sim1+) neuron density from fluorescent intensity at left (see Fig. S6) compared to the count of PT+ neurons as a function of depth and the count of all cortical units recorded in the same sessions. ~6% of all units recorded were identified as tagged in these datasets. The true number of Sim1+ neurons out of the recordable population is not known and may exceed 6%. One estimate of the false positive rate lower bound is to consider a range of anatomical depths at which no Sim1+ somata are thought to be located (<500 microns in depth) indicated by an inflection in fluorescence density and consistent with prior labeling work (*2*). In our imaging experiments we

typically imaged at ~400 µm depth (Fig. 5) and saw little to no somata at this depth for Sim1+ neurons. In this region we recorded 330 single units of which 1 was deemed tagged (str.) or 5 tagged (len.) giving a putative lower bound on false positive rates at 0.3%-1.5%. These estimates amount to removing one or a couple units from distributions on tagged units and there is little impairment to conclusions from such changes. We note that localization of a unit on a Neuropixels probe is not necessarily the somata (proximal dendrite has large extracellular currents during spiking) and thus we do not know that the true positives are 0.

Lastly, we also show a direct comparison between tagged unit average properties and imaged populations with a nominal 0% false positive rate. These are replotted from Figure 3 and 5.
